# Co‐overexpression of VEGF and GDNF in adipose‐derived stem cells optimizes therapeutic effect in neurogenic erectile dysfunction model

**DOI:** 10.1111/cpr.12756

**Published:** 2020-01-13

**Authors:** Wende Yang, Zehong Chen, Xiaolei Ma, Xi Ouyang, Jiafeng Fang, Hongbo Wei

**Affiliations:** ^1^ Department of Gastrointestinal Surgery The Third Affiliated Hospital of Sun Yat‐sen University Guangzhou China

**Keywords:** bilateral cavernous nerve injury, cavernosal fibrosis, erectile dysfunction, GDNF, stem cells, VEGF

## Abstract

**Objectives:**

To evaluate the rapid repair potential of adipose‐derived stem cells (ADSCs) co‐overexpressing VEGF and GDNF on bilateral cavernous nerve injury (BCNI) in rat models. Progressive fibrosis of the penis that occurs shortly after BCNI is a key cause of clinical treatment difficulty of erectile dysfunction (ED). Traditional medications are ineffective for ED caused by BCNI. ADSCs have shown therapeutic effects in animal models, but disappointing in clinical treatment suggests that we should explore optimal treatment of it.

**Materials and methods:**

We extracted ADSCs from rat epididymis. Lentiviral transfection was verified by western blot and immunofluorescence. Thirty‐six SD rats (10 weeks old) were randomly divided into six groups (n = 6 per group): sham surgery, and remaining five BCNI groups transplanted PBS or ADSCs which were genetically modified by vehicle, VEGF (ADSC‐V), GDNF (ADSC‐G), or VEGF&GDNF (ADSC‐G&V) around major pelvic ganglion (MPG). We investigated the therapeutic effects of BCNI rat model which is characterized by ED, penile tissue fibrosis and hypoxia, and lack of nitrogen nerves or vascular atrophy.

**Results:**

Erectile function was almost recovered after 2 weeks of transplantation of ADSC‐G&V, promoted cavernous nerve repair, prevented penile fibrosis and preserving the vascular endothelium, which was significant differences amongst ADSC‐V or ADSC‐G. Moreover, GM‐ADSCs were detected in MPG and penis, indicating that their participation in repair of target organs and transverse nerves.

**Conclusions:**

These promising data indicate that ADSCs co‐overexpressed VEGF and GDNF‐induced synergistic effects, make it a potential tool for recovering of erectile function speedily after BCNI.

## 1 INTRODUCTION

Radical resection of prostate and rectal cancer is the gold standard for the treatment of patients with clinically limited tumours. However, postoperative erectile dysfunction (ED) can result from inadvertent axotomy of the pelvic cavernous nerve (CN) during surgical procedures.^1^, ^2^ Anatomical advances, and the advent of laparoscopic techniques, have reduced CN damage, but postoperative ED is still prevalent.^3^ The CN is a parasympathetic nerve projected from the pelvic ganglion, which lies away from the target organ. After CN damage, it weakens the antagonistic effect on the sympathetic nerve and makes the penile cavernosal smooth muscle diastolic disorder, which is the key reason for the early appearance of ED after CNI.^4^ However, the therapeutic effects of phosphodiesterase type 5 inhibitors (PDE5Is) are limited following CN injury.^5^, ^6^ Furthermore, CN injury (CNI)‐induced ED can progress due to the progressive fibrosis and denervation of the corpus cavernosum.^7^, ^8^ Fibrosis of the corpus cavernosum of the penis could occur 1 week after CNI,^8^ and previous studies have pointed out that the fibrosis response of the penile cavernous cavernosum was closely related to the severity of CN injury and time: 1 week after CNI is acute, 4 weeks after CNI is subacute and 12 weeks after CNI becomes chronic.^9^ Importantly, when CN is successfully repaired in the later period, penile cavernous cavernosum fibrosis may still continue to develop.^7^ Therefore, development of effective strategies to promote rapid repair of CNI that limits or prevented fibrosis of the corpus cavernosum is of great importance. Adipose‐derived stem cells (ADSCs) are more active than bone marrow mesenchymal stem cells (BMMSCs), secrete more autocrine cytokines and immunoregulatory factors, and exhibit lower immunogenicity, which is ideal for cell therapy.^10^, ^11^, ^12^ Adipose tissue is easily obtained by liposuction which has been shown to have more stem cells/progenitor cells than bone marrow tissue, and ADSC has a faster expansion capacity than stem cells derived from other tissues.^13^ In a diabetic rat model of CNI‐induced ED, injection of ADSCs into the corpus cavernosum improved erectile function. This effect was attributed to secreted factors rather than differentiation of ADSCs into different cell types.^14^, ^15^, ^16^ Neovascularization induced by vascular endothelial growth factor (VEGF) can promote Schwann cell (SC) migration‐related nerve regeneration.^17^ Glial cell‐derived nerve growth factor (GDNF) can trigger migration of SCs and increase the survival rate of neurons.^18^ The effects of GDNF did not mimic the bimodal effects of brain‐derived nerve growth factor (BDNF), in which low doses promoted axonal regeneration through the trkB receptor, but high doses inhibited nerve regeneration by acting on the p75 receptor.^19^, ^20^ Although ADSCs do not readily differentiate into vascular endothelial cells, VEGF secreted by ADSCs can support vascular regeneration.^21^ Studies have shown that GDNF secreted by ADSCs can inhibit neuronal apoptosis and promote CN repair.^22^ We hypothesized that CN repair may be mediated by secretion of VEGF and GDNF.^23^ To evaluate the optimized therapeutic effects of ADSCs, we used lentivirus to induce overexpression of VEGF and GDNF and transplanted these cells in the vicinity of the major pelvic ganglion (MPG). The results showed that ADSC‐G&V group significantly improved erectile function in bilateral CNI (BCNI) rats and preserved the corpus cavernosum microstructure compare with other types of ADSCs. Furthermore, GM‐ADSC‐treated rats showed significant downregulation of the p‐LIMK2/p‐cofilin pathway, which suggested that BCNI‐induced fibrosis in the corpus cavernosum may be prevented. The results of this study may indicate a new clinical strategy to speedily ameliorate CNI‐induced ED.

## 2 METHODS AND MATERIALS

### 2.1 Animals

Two‐week‐old male Sprague‐Dawley (SD) rats (weighing approximately 50‐100 g body weight) and 10‐week‐old male SD rats (weighing approximately 250‐300 g body weight) were obtained from the Experimental Animal Center of Sun Yat‐sen University. These rats were used to isolate ADSCs and to generate a BCNI model, respectively. All rats used in this study were housed in the Animal Experimental Center of the Third Affiliated Hospital of Sun Yat‐sen University, exposed to a 12‐hour light/dark cycle and maintained at 22 ± 2°C with water and food available ad libitum. All procedures were approved by the Ethics Committee of the Institutional Animal Care and Use Subcommittee of the Third Hospital of Sun Yat‐sen University. Thirty‐six SD rats (10 weeks old) were randomly divided into six groups (n = 6 per group). One group underwent sham surgery, and each of the remaining five groups underwent BCNI. The groups that underwent BCNI received the following treatments at the MPG: PBS, non‐genetically modified ADSC, ADSC overexpression of VEGF (ADSC‐V), ADSC overexpression of GDNF (ADSC‐G), or ADSC overexpression of VEGF and GDNF (ADSC‐G&V).

### 2.2 Cell preparation and identification

Adipose‐derived stem cells were isolated from epididymal adipose tissue of 2‐week‐old SD rats as previously described.^24^ Cells were resuspended in Dulbecco's modified Eagle's medium (DMEM) supplemented with 10% foetal bovine serum and incubated in a culture flask at 37°C in a humidified atmosphere containing 5% CO_2_. Before lentiviral transduction, ADSCs were stained with fluorescein isothiocyanate‐labelled specific antibodies against CD34, CD11b, CD29, CD44a, CD45 or CD90, then analysed using flow cytometry. As described previously, the ability of ADSCs to differentiate into adipocytes and osteoblasts was examined using a special medium.^25^ The medium was changed every 3 days. After 2 weeks, osteoblasts were identified using alizarin red S staining. After four additional weeks, adipocytes were identified using oil red O staining. All cells were used at passages 3‐5.

Two‐week‐old SD rats were anaesthetized and killed. The bilateral sciatic nerves were removed and transferred to a sterile table. The outer membrane and connective tissue were removed on ice in Hank's balanced salt solution (HBSS) +1% penicillin/streptomycin. The nerve bundle was extracted using sterile forceps and cut into 3‐5 mm pieces, transferred to a 6‐well plate and incubated in DMEM high glucose medium +10% FBS at 37°C and 5% CO_2_. Cell migration from nerve fragments was evaluated after incubating for 1 week. When the cells reached confluence, they were detached and transferred to a T25 flask. Schwann cells (SCs) were isolated using 5 µg/mL cytarabine to remove rapidly proliferating fibroblasts. Primary SCs at passage 2 were immunofluorescence stained for S100β to demonstrate cellular marker.

### 2.3 Construction lentivirus and transfection

Lentiviral constructs expressing rat VEGF and GDNF were prepared by Hanbio Biotechnology (shanghai) using HBLV‐r‐VEGFA‐3xflag‐PFP‐PURO and HBLV‐r‐GDNF‐3xflag‐BSD‐GFP, respectively. The sequence‐verified constructs were transfected into human embryonic kidney (HEK) 293T cells using Lipo2000 for lentiviral packaging. The virus supernatant was collected at 72 hours, then filtered through a 0.45 µm filter, and centrifuged at 72 000 *g* for 12 minutes at 4°C in a 40 mL ultracentrifuge tube. The virus pellet was resuspended in 500 μL of fresh medium, the virus titre was determined using a serial dilution method, and the virus was stored at −80°C.

Adipose‐derived stem cells at passage 2 were co‐transduced or single‐transduced with lentiviral constructs at a multiplicity of infection (MOI) of 100. Adipose‐derived stem cells transfected with VEGF or GDNF were screened with 2 μg/mL puromycin and 15 μg/mL blasticidin, respectively. After 1 week of screening, red and green fluorescence were observed using an immunofluorescence microscope to confirm successful transfection of ADSCs. In addition, the expression of GDNF and VEGF was detected using western blot. Following characterization, the cells were collected and resuspended in PBS for use in animal experiments.

### 2.4 Preparation of cell supernatants

Five different types of cells (ADSCs, vehicle, ADSC‐V, ADSC‐G and ADSC‐G&V) were cultured in 6‐well plates (10^5^ cells/well). When the cells reached 90% confluence, the medium was replaced with 1 mL of serum‐free medium, and the cells were incubated for 24 hours. The supernatant was collected after centrifugation at 12 000 *g* for 10 minutes and stored at −80°C.

### 2.5 Human umbilical vein endothelial cell tube formation assay

The human umbilical vein cell line, EA. hy926 (HUVEC), was gifted by Professor Gexiu Liu, Institute of Hematology, Jinan University. Tube formation was evaluated by culturing HUVEC on BD Matrigel (BD Biosciences). After incubating the wells with 80 μL of Matrigel for 1 hour, the HUVEC were resuspended in the cell supernatants of the above different cell sources and DMEM medium alone as negative control (NC) into 96‐well plates (5000 cells per well), then the number of tube‐like structures were analysed 4 hours later. Quantitative analysis based on the number of lumens in each high‐power field.

### 2.6 Chemotaxis of primary Schwann cells

Evaluation of chemotaxis of primary SCs was performed using an 8‐μm pore membrane filter (PIEP12R48, Millipore). Each upper chamber was filled with serum‐starved primary SCs (2 × 10^5^ cells, 300 μL/well), and each lower chamber was filled with cell supernatant (500 μL) from different cells. After incubating for 10 hours at 37°C in a humidified atmosphere containing 5% CO_2_, the remaining cells on the upper surface were gently wiped with a cotton swab, and the filter was fixed and stained with 0.1% crystal violet. Cells that migrated to the lower surface were counted using a microscope.

### 2.7 Establishment of the BCNI model and cell transplantation

To generate the BCNI rat model, rats were weighed and anaesthetized with 2.5%‐3% isoflurane. The nerve crush site was located 2‐5 mm distal to the MPG, and the injury was induced as previously described.^16^ The sham group underwent an identical procedure, but the nerves were not crushed. Different types of cell‐fibrin scaffolds (1.5 × 10^6^ cells, 100 μL per rat) were prepared according to the instructions provided with the Porcine Fibrin Sealant Kit (Hangzhou Puji Medical Technology Development Co. Ltd.). The cell‐fibrin scaffold mixture was implanted around the MPG as previously described.^26^

### 2.8 Erectile function assessment

Under deep anaesthesia, the rats were placed on a warm pad and a midline incision was made to expose the right carotid artery from the neck to the upper chest. A heparinized 24‐gauge silastic cannula was inserted to measure the mean arterial blood pressure (MAP). The MPG and cavernous nerves were exposed through the midline incision. The corpus cavernosum was intubated with a heparinized (250 U/mL) 25‐gauge butterfly needle by insertion at the crura. The cannula was connected to the BL‐420 biological function system (Chengdu Taimeng Technology Ltd) for continuous evaluation and recording of ICP. The stimulus parameters were 1.5 mA, 20 Hz, pulse width 0.2 ms and duration 50 seconds.^27^ The three maximum increases in ICP per side for each animal were chosen for statistical analysis of the mean ICP. The penis, MPG and distal cavernous nerves were harvested for histological analysis and western blotting. The rats were killed by anaesthesia overdose.

### 2.9 Histological examination

A portion of the MPG and penile mid‐shaft tissue was fixed immediately after harvesting for immunofluorescence staining, as described previously.^16^ The MPG sections were incubated with a primary antibody against Neurofilament‐H (1:400, CST, RMdo20), and the penile tissues were incubated with anti‐nNOS (1:200, Abcam, ab1376), anti‐Desmin (1:200, Abcam, ab32362), anti‐HIF‐1α (1:50, Abcam, ab179483), anti‐RECA‐1 (1:50, Abcam, ab9774), anti‐VEGF (1:100, Affinity, AF5131) and anti‐GDNF (1:100, Servicebio, gb11403). DyLight 488‐ and 556‐conjugated antibodies were used as secondary antibodies (Invitrogen, 1:500), and nuclei were stained with DAPI (0.5 μg/mL; Invitrogen). Images were visualized and acquired using a confocal laser scanning microscope (Zeiss LSM 710).

The MPG was harvested and fixed in 4% phosphate‐buffered paraformaldehyde, embedded in paraffin (BioCheck Laboratories) and sectioned (5 μm) for immunohistochemistry analysis. For immunohistochemical staining, endogenous peroxidase activity in MPG sections was inactivated using methanol containing 0.3% H_2_O_2_, and antigen recovery was performed by incubation with 0.1% trypsin for 30 minutes at 37°C, followed by two rinses with PBS. The sections were blocked with 1% bovine serum albumin (BSA) for 2 hours at room temperature and incubated for 1 hour with primary antibodies against S100β (1:3000, Abcam, ab14849), VEGF (1:500, Servicebio, gb11034b) and GDNF (1:500, Servicebio, gb11403). After washing, the sections were incubated with HRP (1:200 dilution, Abcam) for 1 hour at room temperature, then washed twice with PBS. All sections were counterstained with haematoxylin to stain nuclei, placed on coverslips and observed using a microscope. The immune score was the percentage of positive cells multiplied by the stain intensity. The scale was as follows: no positive cells (0 points), less than 10% (1 point), positive cells between 10% and 50% (2 points), positive cells between 50% and 80% (3 points), positive cells greater than 80% (4 points). Staining intensity was scored as follows: no staining (0 points), mild staining (1 point), moderate staining (2 points) and deep staining (3 points). Masson trichrome staining was used to assess the ratio between smooth muscle and collagen in the corpus cavernosum, as previously described.^27^ Quantitative analysis of images was performed using Image J k 1.45 (National Institutes of Health).

### 2.10 Western blotting

Tissues and cells were lysed using RIPA (Sigma‐Aldrich) buffer containing a mixture of protease inhibitors for western blot analysis. Protein concentrations in tissues and cells lysates were determined using a BCA (Biosharp, BL521A) kit. Thirty micrograms of total protein were subjected to sodium dodecyl sulphate polyacrylamide gel electrophoresis, then transferred to polyvinylidene fluoride membranes. The membranes were blocked with 5% BSA and incubated overnight at 4°C with primary antibodies against p‐LIMK2 (1:1000, CST, #3841), LIMK2 (1:2000, Abcam, ab45165), p‐cofilin (1:1000, CST, #3313), cofilin (1:1000, Abcam, ab124979), VEGF (1:500, Affinity, AF5131), GDNF (1:5000, Abcam, ab176564) and β‐actin (1:1000, CST, #4970) or GAPDH (1:5000, Abcam, ab181602). After incubating with secondary antibodies at room temperature, images were acquired using a Tanon 5200 (Tanon Science & Technology Co, Ltd), and the average integrated density of the bands was analysed using Image J K1.45 (National Institutes of Health).

### 2.11 Statistical analysis

Graphs were plotted using GraphPad Prism (version 5) (GraphPad Software) and expressed as means ± standard deviations. Comparisons between two groups were performed using Student's unpaired *t* test. Differences amongst more than two groups were determined using one‐way ANOVA followed by the S‐N‐K test using SPSS 16.0 software (SPSS Inc). Quantitative analysis of immunohistochemistry was performed using the Kruskal‐Wallis *H* test. *P < *.05 was considered statistically significant.

## 3 RESULTS

### 3.1 GM‐ADSCs produced more VEGF and GDNF

**Figure 1:**
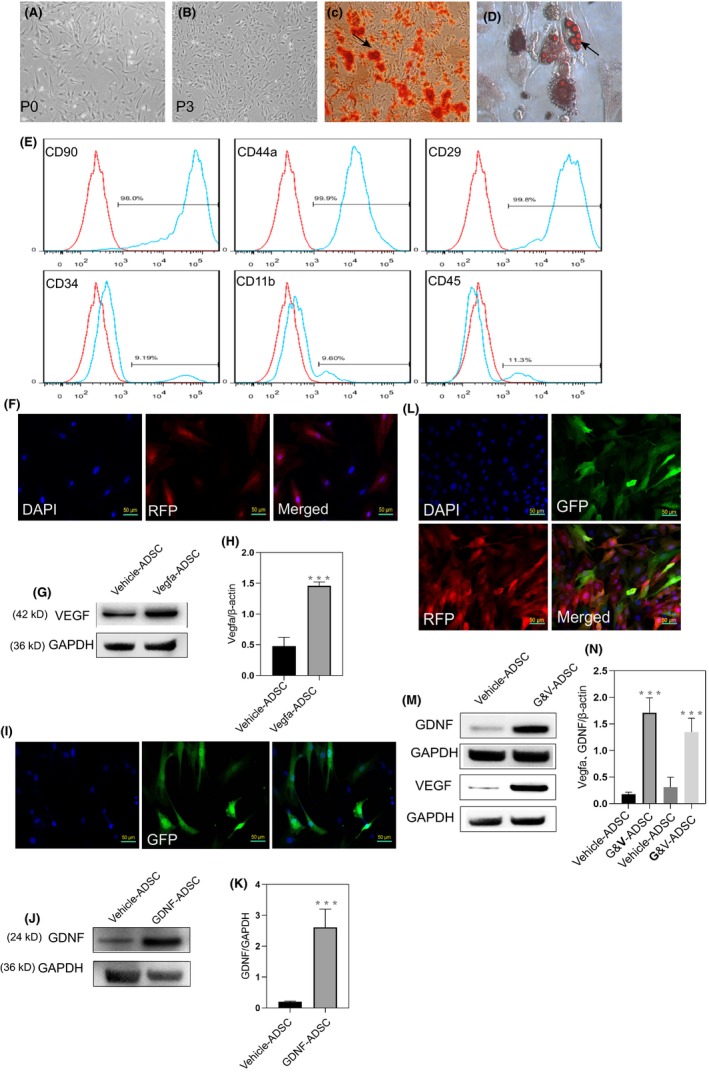
Genetically modified ADSCs produced more VEGF and GDNF. A,B, Morphology of ADSCs in passage 0 and passage 3. C,D, Adipose‐derived stem cells exhibited pluripotency after induction, as evidenced by the presence of typical phenotype of osteocytes (stained with Alizarin Red S) and the typical phenotype of adipocytes (stained with Oil Red O). E, Flow cytometry showed the ADSCs expressed more stem cell markers (CD90, CD44a and CD29), but less haematopoietic and endothelial markers (CD34, CD11b and CD45). F, Immunofluorescence analysis of the expression of RFP in transfected ADSCs. G,H, Western blot analysis showed markedly increased the levels of VEGF in transfected ADSCs. I, Immunofluorescence analysis of the expression of GFP in transfected ADSCs. J,K, Western blot analysis showed increased the levels of GDNF in transfected ADSCs. L, Immunofluorescence of ADSCs that co‐expressed RFP and GFP. M,N, Western blot analysis of ADSCs co‐transfected with VEGF and GDNF. All values are represented as the mean ± SD from three independent experiments, each with three replicates. Statistically significant differences from the control group are denoted as follows: ***P < .001, **P < .01 and *P < .05 (independent samples *t* test)

Primary ADSCs were isolated from epididymal adipose tissue of 2‐week‐old Sprague‐Dawley rats cultured in vitro. Spindle morphology and fibroblast‐like morphology were observed at passage 1, and these characteristics were more pronounced at passage 3 (Figure 1A,B). To determine the pluripotency of the ADSCs, we evaluated passage 3 cells for osteogenic and adipogenic differentiation. The results showed that the cells were positive for alizarin red staining 2 weeks after induction, and positive for oil red O staining 4 weeks after induction (Figure 1C,D). To further verify that the cells were ADSCs, we identified surface markers using flow cytometry. The results showed that the following surface stem cell markers of ADSCs were expressed: CD90 (98%), CD44a (99.9%), and CD29 (99.8%). In contrast, the following haematopoietic markers were expressed in a lower percentage of cells: CD34 (9.1%), CD11b (9.6%) and CD45 (11%) (Figure 1E).

Fluorescence microscopy showed that most cells expressed red fluorescent protein, which indicated successful transduction. Furthermore, western blot analysis showed that the expression of VEGF was significantly higher in ADSCs transfected with VEGF than that in vehicle‐treated ADSCs (Figure 1F,G,H). Expression of green fluorescent protein demonstrated that the expression of GDNF was significantly higher in ADSCs transfected with GDNF than that in vehicle‐treated ADSCs (Figure 1I,J,K). Transfection with VEGF and GDNF resulted in red and green fluorescence, which indicated successful co‐transfection. Western blot results confirmed that VEGF and GDNF were expressed at higher levels in GM‐ADSCs than in vehicle (Figure 1L,M,N).

### 3.2 ADSCs transfected with VEGF and GDNF enhanced tubule formation of HUVECs and chemotaxis of primary SCs in vitro

**Figure 2:**
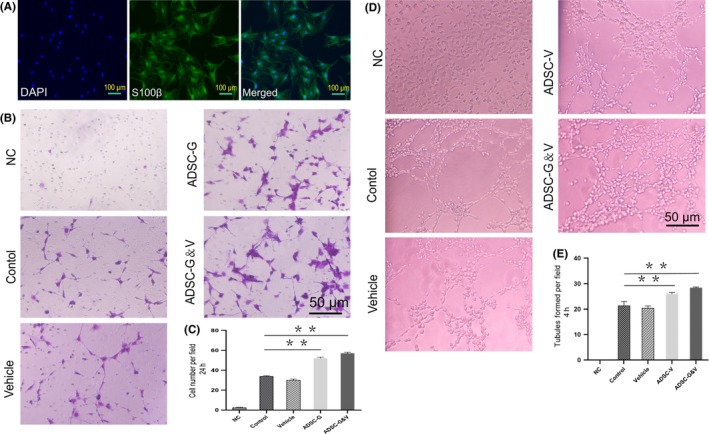
Adipocyte‐derived stem cells transfected with VEGF and GDNF enhanced tubule formation of HUVECs and chemotaxis of primary SCs, respectively. A, Isolation and identification of primary SCs from rat sciatic nerves (passage 2). B, Schwann cell chemotaxis in response to different cell culture supernatants by crystal violet staining and visualization using an inverted microscope. C, Quantification of migrated cells. D, Tubule formation of HUVECs cultured with different supernatants. E, Quantitative analysis of tubular formation of HUVECs. Each bar depicts the mean ± SD (***P < .001, **P < .01 and *P < .05, n = 3); one‐way ANOVA followed by the S‐N‐K test

The chemotactic effect of GDNF on SCs is a critical step in peripheral nerve regeneration.^18^ To determine whether ADSCs that overexpressed GDNF promoted SC chemotaxis, we isolated SCs from the rat sciatic nerve and verified that they were SCs by S100β immunostaining^28^ (Figure 2A). Primary SCs at passage 2 were co‐cultured with supernatants of different cultured cell types. The ADSC‐G group and the ADSC‐G&V group induced stronger SC chemotaxis than the control and vehicle groups. There was no difference in the chemotactic effect induced by ADSC‐G and ADSC‐G&V (Figure 2B,C), which suggested that overexpression of GDNF in ADSC was responsible for SC chemotaxis.

The ability of cells or signalling factors to induce angiogenesis can be evaluated by tubule formation of HUVECs. Supernatants collected from different types of cells were co‐cultured with HUVECs. Culture supernatants from ADSC‐V and ADSC‐G&V promoted more extensive tubule formation than culture supernatants from the control and vehicle groups. No differences in tubule formation were observed between the ADSC‐V and ADSC‐G&V groups (Figure 2D,E). These results indicated that ADSC overexpression of VEGF and GDNF enhanced SC chemotaxis and HUVEC tubule formation, which indicated that overexpression of these factors resulted in the predicted biochemical effects.

### 3.3 Expression of VEGF and GDNF in the MPG and penis following GM‐ADSCs transplantation

**Figure 3:**
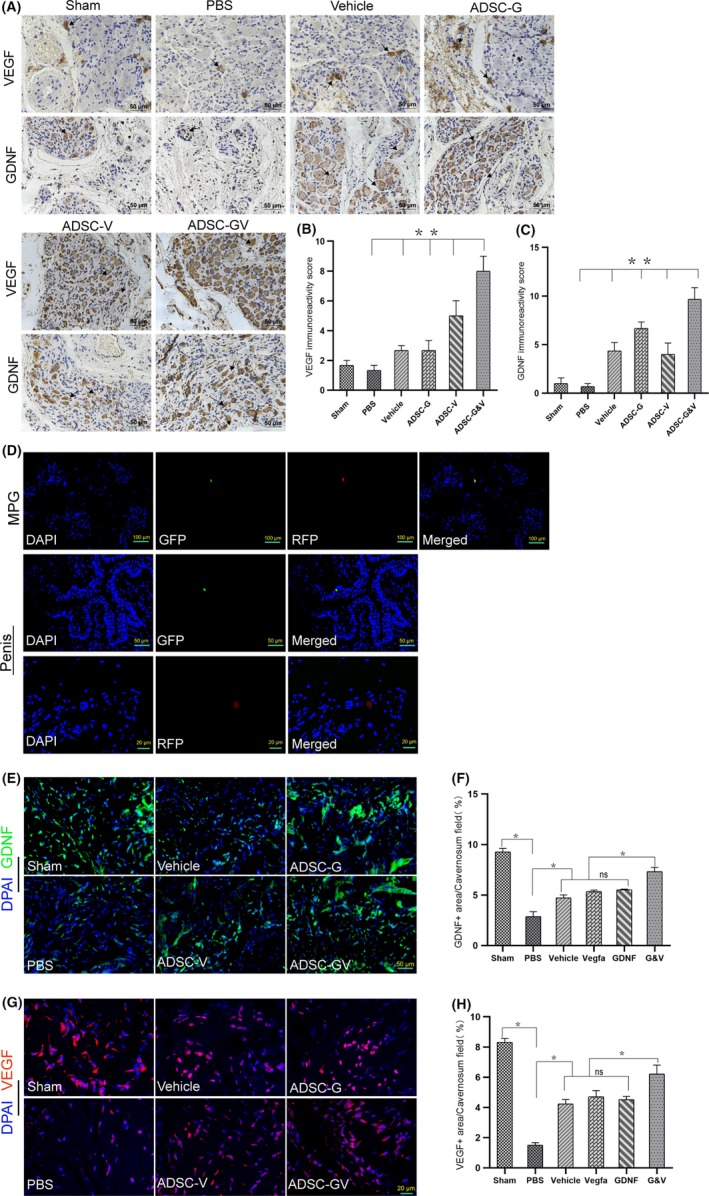
The expression of VEGF and GDNF in the MPG and penis after transplantation of GM‐ADSCs. A, Immunohistochemical staining of rat MPG tissue cross‐sections from each animal in the respective groups using specific VEGF and GDNF antibodies. Arrows indicate typical immune‐positive cells. B,C, Immunohistochemical score was obtained by analysing the staining intensity and positive rate of VEGF and GDNF. D, After 14 d, transfected ADSCs were visible in the MPG and corpus cavernosa. E, Representative immunofluorescence staining of GDNF (green) in a penile mid‐shaft specimen 2 wk after BCNI and treatment. F, Quantitative analysis of the GDNF‐positive area. G,H, Representative immunofluorescence of VEGF in a penile mid‐shaft specimen, and quantitative analysis of the VEGF immunofluorescence‐positive area. Data are depicted as the mean ± SD from n = 6 animals per group (*P < .05). Immunofluorescence was analysed using one‐way ANOVA followed by the S‐N‐K test. Quantitative analysis of immunohistochemistry was performed using the Kruskal‐Wallis *H* test

Immunohistochemical analysis was used to investigate the expression of VEGF and GDNF in the MPG 2 weeks after transplantation of GM‐ADSCs. The data showed that the expression of VEGF differed in the MPG amongst the different groups (*P* < .05). In addition, the expression of VEGF in the ADSC‐G&V group was significantly higher than that in the other groups. The expression of GDNF in the MPG differed significantly amongst the different groups (*P* < .05). Furthermore, the expression of GDNF in the ADSC group was significantly higher than that in the other groups, including the ADSC‐G group (Figure 3A,B,C). Two weeks after transplantation, GM‐ADSCs were present in the MPG and in the penis (Figure 3D). To evaluate the expression of VEGF and GDNF in the penis, immunofluorescence was performed on penile mid‐shaft specimens from each experimental group. The results showed that the levels of VEGF and GDNF in the ADSC‐G&V group were significantly higher than those in the other groups (*P* < .05). No significant differences in the expression of VEGF and GDNF were observed amongst the vehicle, ADSC‐V and ADSC‐G groups (Figure 3E,F,G,H).

### 3.4 Treatment with GM‐ADSCs improved erectile function in BCNI rats

**Figure 4:**
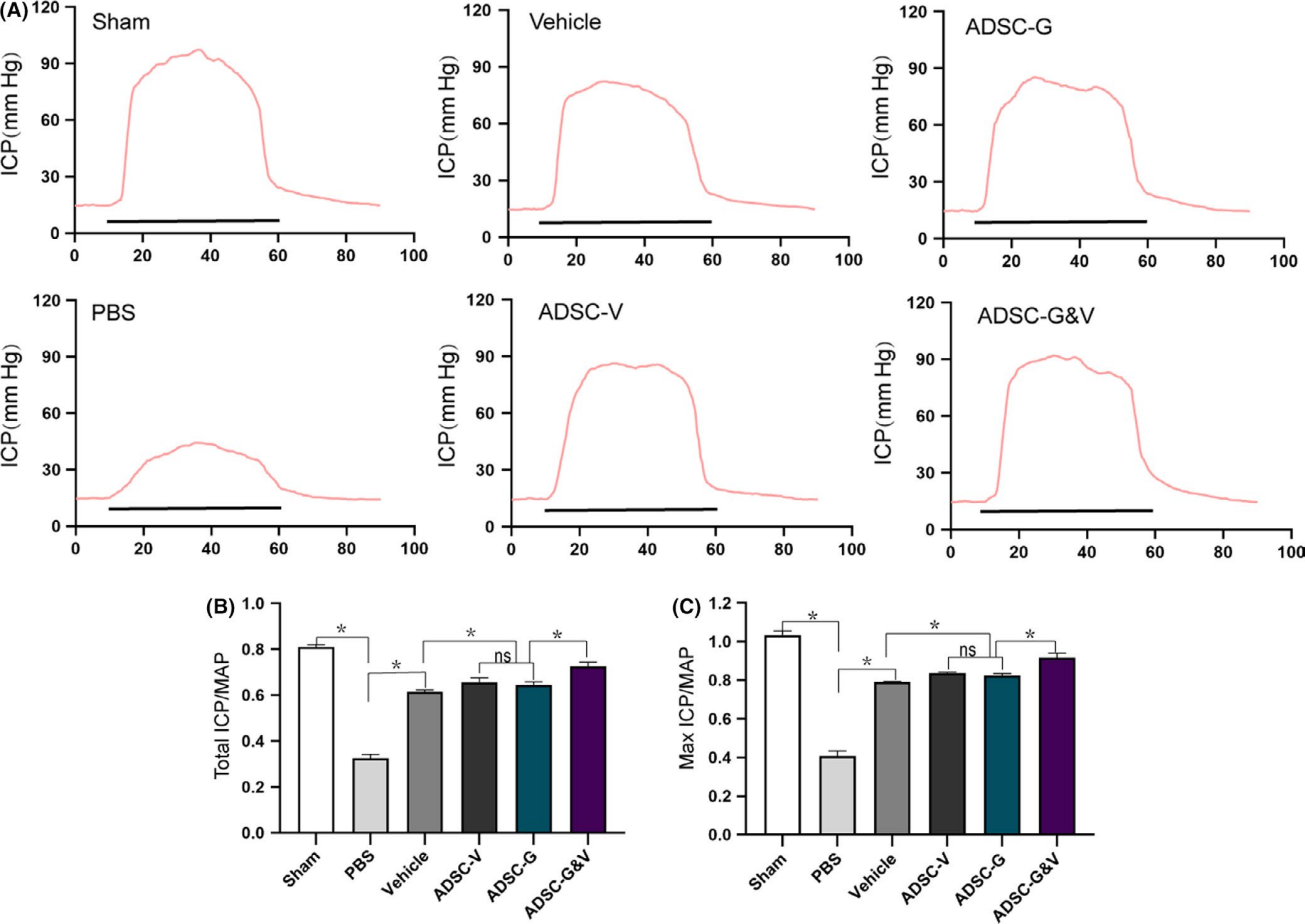
Treatment with GM‐ADSCs improved erectile function in BCNI rats. A, Changes in intracavernous pressure (ICP) by electrical stimulation of cavernous nerve (CN). Representative ICP dynamic response of six experimental animals. The black bar represents the duration of electrical stimulation (50 s). B,C, Quantitative analysis of total ICP (under the curve)/average arterial pressure (MAP) and Max ICP/MAP. All values depict as mean ± SD from n = 6 animals per group (−*P < .05); one‐way ANOVA followed by the S‐N‐K test

Changes in the ratio of intracavernous pressure (ICP) to mean arterial pressure (MAP) induced by electrical stimulation of the corpus cavernosum can reflect penile erectile function in rats. The sham group showed normal ratios of total ICP (area under the curve) to MAP and Max ICP to MAP. In contrast, the BCNI group showed significantly lower ICP to MAP and Max ICP to MAP ratios than the sham group (*P* < .05), which indicated that ED was induced. Treatment with different GM‐ADSCs promoted differing degrees of improvement of erectile function. ADSC‐GV group had statistically differences in functional improvement compared with vehicle, ADSC‐V and ADSC‐G groups, in addition, the improvements of ADSC‐V and ADSC‐G were also statistically different compared with the vehicle group (*P* < .05) (Figure 4A,B,C).

### 3.5 Transplantation of GM‐ADSCs increased endothelial content and improved hypoxia in the corpus cavernosum

**Figure 5:**
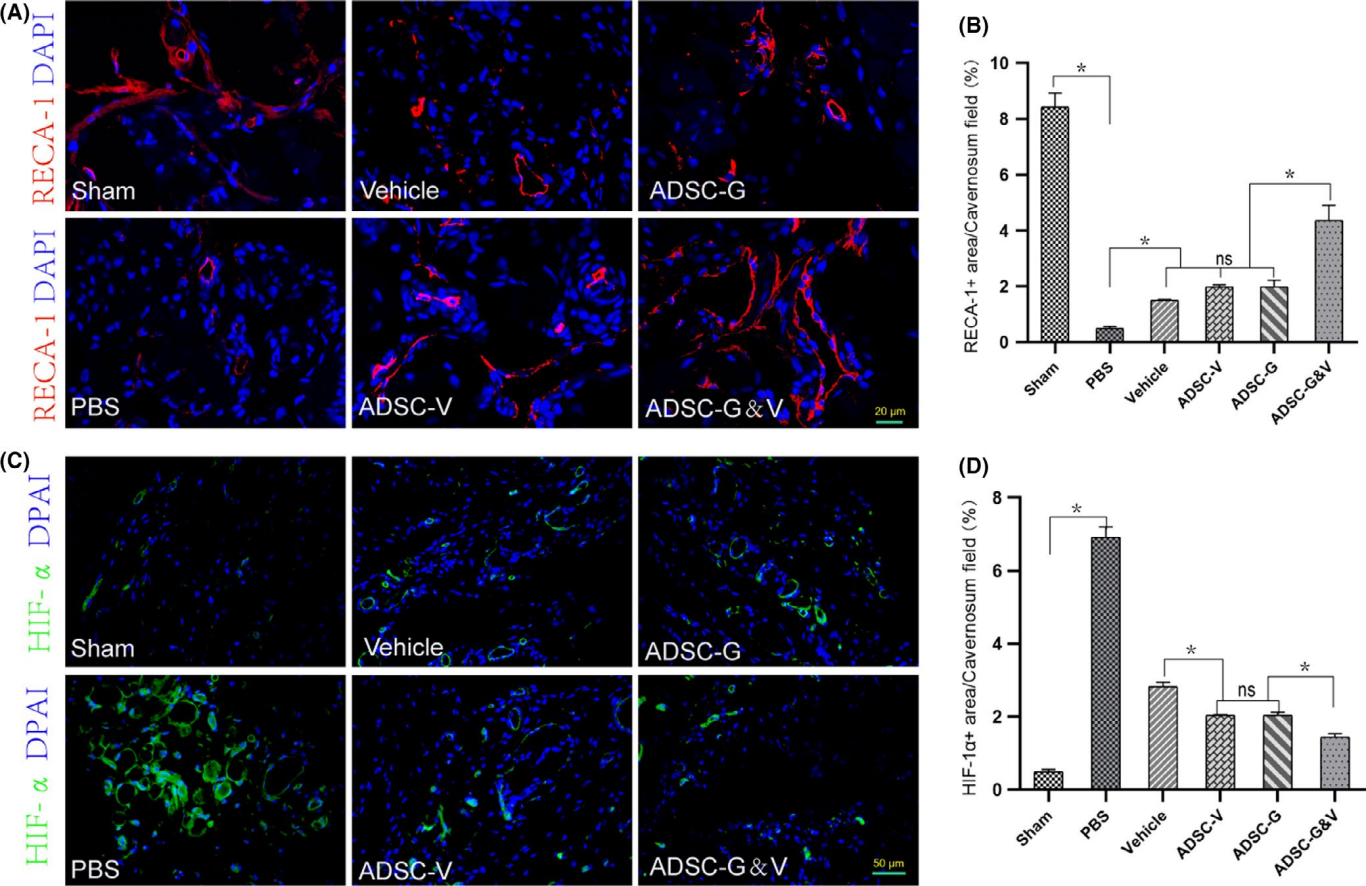
Transplantation of GM‐ADSCs increased endothelial content and improved hypoxia in the corpus cavernosum. A, Representative images of immunofluorescence staining of RECA‐1 (red) and with DAPI (4′,6‐diamidino‐2‐phenylindole) in a penile mid‐shaft specimen 2 wk after BCNI and treatment. B, Quantitative analysis of the RECA‐1 immunofluorescence‐positive area. C, Representative images of immunofluorescence staining of HIF‐1α (green) in a penile mid‐shaft specimen. D, Quantitative analysis of the HIF‐1α immunofluorescence‐positive area. Data are depicted as the mean ± SD from n = 6 animals per group (*P < .05). Immunofluorescence was analysed using one‐way ANOVA followed by the S‐N‐K test

Penile erection is a complex process coordinated by multiple tissues. Functional endothelial tissue in the cavernosum is critical to this process and can be reflected by expression levels of RECA‐1.^29^ Two weeks after BCNI, the PBS group exhibited low expression of RECA‐1 compared with the sham group, as determined using immunofluorescence (*P* < .05). The expression of RECA‐1 was increased to varying degrees in the GM‐ADSCs groups, and transplantation of ADSC‐G&V resulted in the greatest effect which was significant differences amongst vehicle, ADSC‐V and ADSC‐G (*P* < .05) (Figure 5A,B). Vascular endothelial function is closely related to degree of hypoxia. We found that the expression of HIF‐1α in the PBS group was significantly higher than that in the sham group (*P* < .05). In addition, the expression of HIF‐1α was decreased in the GM‐ADSCs groups, this decrease was greatest in the ADSC‐G&V group, ADSC‐G and ADSC‐V groups also improved significantly compared to vehicle group (*P* < .05) (Figure 5C,D).

### 3.6 Transplantation of GM‐ADSCs prevented fibrosis of the corpus cavernosum and increased cavernosal smooth muscle content

**Figure 6:**
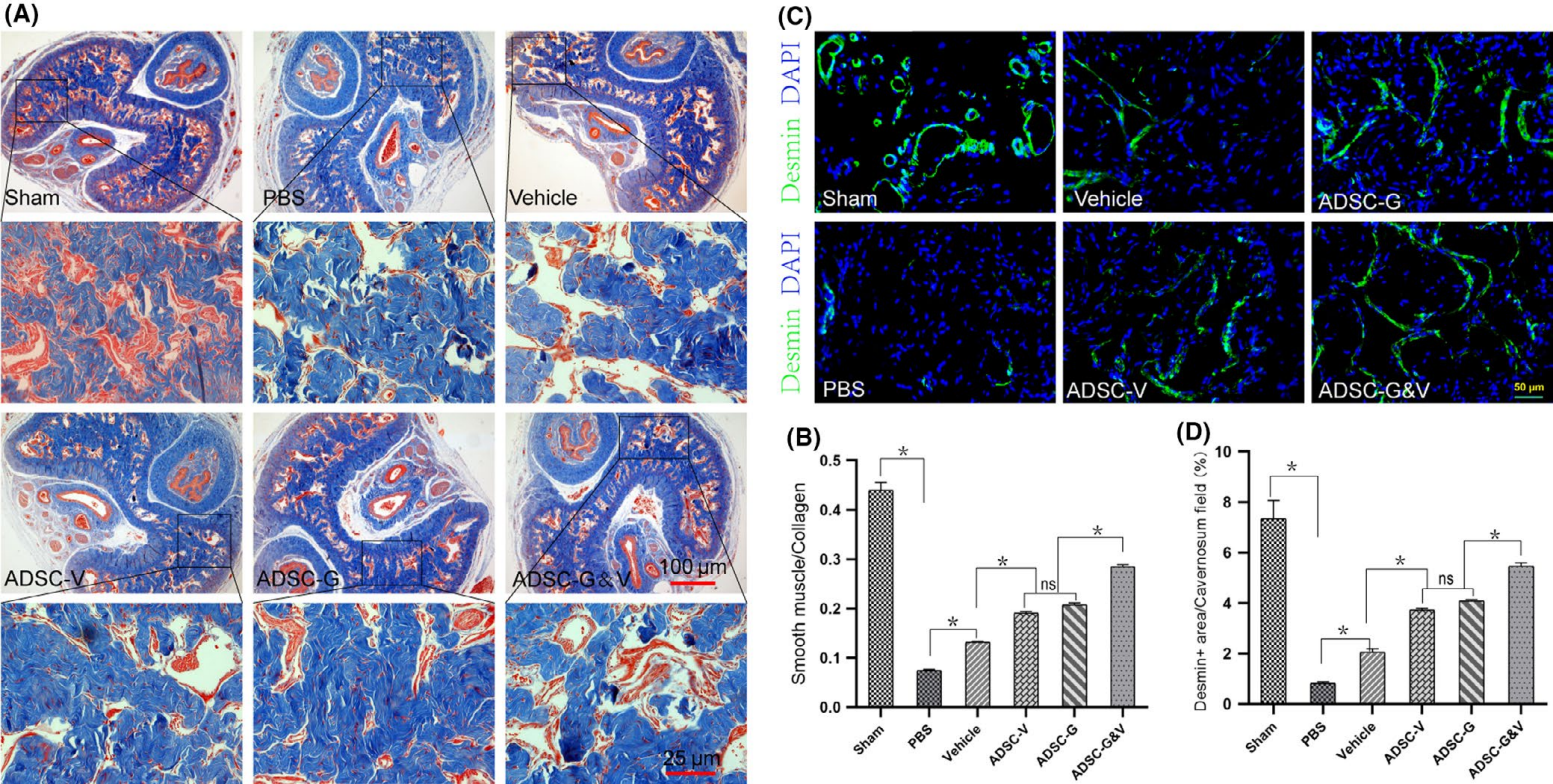
Genetically modified ADSCs prevented fibrosis in the corpus cavernosum and increased cavernosal smooth muscle content. A, Representative images of Masson trichrome staining in a penile mid‐shaft specimen from each experimental group. Smooth muscle and collagen were stained red and blue, respectively; B, Quantitative analysis of Masson trichrome staining results. C, Representative images of desmin (green) immunofluorescence staining in a penile mid‐shaft specimen. D, Quantitative analysis of the desmin immunofluorescence‐positive area. Data are depicted as the mean ± SD from n = 6 animals per group (*P < .05). Immunofluorescence was analysed using one‐way ANOVA followed by the S‐N‐K test

Corpus cavernosum fibrosis caused by CNI is an important mechanism of development of ED.^30^ Using Masson trichrome staining, we found that the proportion of smooth muscle (SM) to collagen in the sham group was higher than that in the PBS group (*P* < .05). Compared with the PBS group, the GM‐ADSCs groups showed significantly increased ratios of SM to collagen, and the ADSC‐G&V group showed the greatest improvement (*P* < .05). There were no significant differences between the ADSC‐V and ADSC‐G groups, but both improved the SM to collagen ratio compared to the vehicle group (Figure 6A,B). We used desmin immunofluorescence staining of the corpus cavernosum to further evaluate SM content. The results showed that the expression of desmin in the sham group was significantly higher than that in the PBS group (*P* < .05). In addition, desmin expression in the ADSC‐G&V group was similar to that in the sham group and differed from that in the other GM‐ADSCs groups (Figure 6C,D).

### 3.7 Transplantation of GM‐ADSCs promoted nerve regeneration

**Figure 7:**
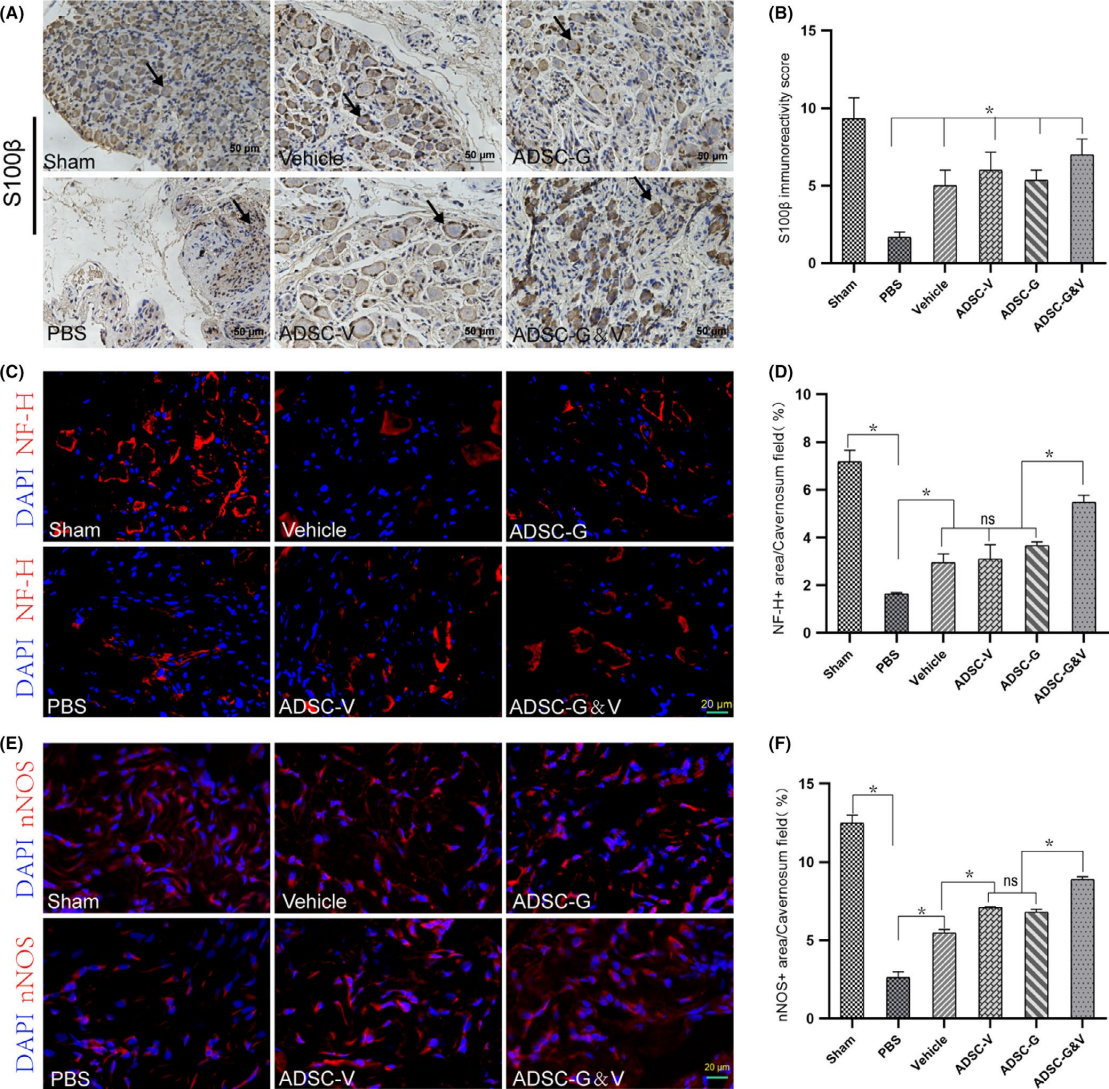
Genetically modified ADSCs promoted nerve regeneration. A, Immunohistochemical staining of rat MPG tissue cross‐sections using specific a S100β antibody. Representative images from each experimental group are shown. B, Quantitative analysis of the immunohistochemistry results for S100β. C, Neurofilament‐H (NF‐H) (red) immunofluorescence staining of MPG tissue cross‐sections from rats in each experimental group. D, Quantitative analysis of immunofluorescence results for NF‐H. E, Neuronal nitric oxide synthase (nNOS) immunofluorescence staining in a penile mid‐shaft specimen. Neuronal NOS positive fibres were stained red. F, Quantitative analysis of immunofluorescence results for nNOS. Data are depicted as the mean ± SD from n = 6 animals per group (*P < .05). Immunofluorescence was analysed using one‐way ANOVA followed by the S‐N‐K test. Quantitative analysis of immunohistochemistry was performed using the Kruskal‐Wallis *H* test

We performed S100β immunohistochemical staining on rat MPG tissues to evaluate in vivo nerve regeneration. The results showed that there were significant differences amongst the six experimental groups (*P* < .05). The expression of S100β in the ADSC‐G&V group was significantly higher than that in the other GM‐ADSC groups (Figure 7A,B). The expression of neurofilament‐H (NF‐H) in the MPG was significantly lower in the PBS group than that in the sham group (*P* < .05). The expression of NF‐H in the ADSC‐G&V group was greater than that in the other three ADSC groups, but there were no significant differences amongst these three groups (Figure 7C,D). The expression of nNOS in the corpus cavernosum was evaluated using immunofluorescence staining 2 weeks after GM‐ADSC transplantation. The data showed that the expression of nNOS in the ADSC‐G&V group was higher than that in the PBS group (*P* < .05). The expression of nNOS in the ADSC‐V and ADSC‐G groups was greater than that in the vehicle group, but there were no differences between the ADSC‐V and ADSC‐G groups (Figure 7E,F).

### 3.8 GM‐ADSCs prevented fibrosis of cavernosal tissues after BCNI by downregulating the p‐LIMK2/p‐cofilin pathway

**Figure 8:**
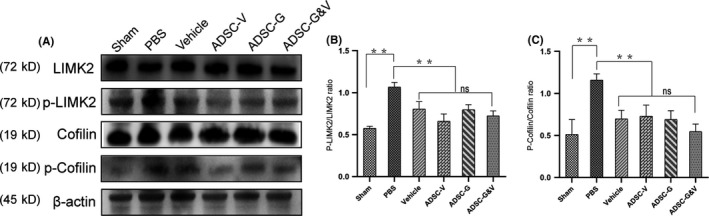
Genetically modified ADSCs prevented fibrosis of cavernosal tissues after BCNI by downregulating the p‐LIMK2/p‐cofilin pathway. A, Representative images of western blots for phosphorylated LIMK2 (p‐LIMK2), LIMK2, phosphorylated cofilin (p‐cofilin) and cofilin in cavernosal tissues from each group. B,C, Quantitative analysis using the ratio of phosphorylated protein to total protein. Data are depicted as the mean ± SD from n = 6 animals per group (**P < .01 and *P < .05); one‐way ANOVA followed by the S‐N‐K test

Reversing fibrosis in the corpus cavernosum is critical to treatment of ED. As shown in Figure 8, the expression of p‐LIMK2 was highest in the PBS group. In contrast, the expression of p‐LIMK2 was lower in the sham group and in the GM‐ADSCs group compared to that in the PBS group (*P* < .05). The expression of LIMK2 did not differ amongst any of the groups. Phosphorylated cofilin, a downstream effector of p‐LIMK2, showed an expression pattern similar to that of p‐LIMK2. The expression of p‐cofilin in the sham group and in the GM‐ADSC groups was significantly lower than that in the PBS group. The expression of cofilin did not differ amongst any of the groups (Figure 8A). Analysis of western blot bands by densitometry showed that the ratio of p‐LIMK2 to LIMK2 in the PBS group was significantly higher than that in the sham group and the GM‐ADSCs groups (*P* < .05). The ratio of p‐cofilin to cofilin differed amongst the different groups and was significantly higher in the PBS group than in the other five groups (*P* < .05) (Figure 8B,C).

## 4 DISCUSSION

Functional penile smooth muscle (SM), intact endothelium and characteristic nitrogen nerves coordinate to ensure normal penile erectile function.^31^ The CN is the main nerve that innervates the penis, and temporary nerve block may occur after inadvertent injury during pelvic surgery, resulting in ED.^2^ However, persistent penile denervation can result in changes in the corpus cavernosum, which typically manifests as penile fibrosis.^32^ Previous studies using rat models of BCNI have shown that penile fibrosis occurs early in the post‐injury period and progresses over time.^33^ However, after structural changes such as progressive fibrosis of the cavernous tissue, improvement of neurological apraxia is not likely to result in recovery of erectile function.^32^ These results suggested that timely functional repair of CNI is critical to successful treatment of postoperative ED. Phosphodiesterase 5 inhibitors are commonly used to treat ED, but are not effective for treatment of ED caused by CNI.^5^, ^6^ In rodent BCNI models, there is a general consensus that MSC‐based treatment can improve erectile function to varying degrees.^23^, ^34^, ^35^ However, in nine patients with diabetes‐related ED who received MSC treatment in South Korea, seven patients experienced a partial increase in penile stiffness, but could not complete vaginal penetration,^36^ which suggested that optimization of MSC treatment is necessary. In this study, we overexpressed VEGF and GDNF in ADSCs and transplanted these cells around the MPG of rats that underwent BCNI. Functional and morphological observations after 2 weeks showed that the ADSC‐G&V group promoted CN repair, resulting in improved erectile function and corpus cavernosum structure.

A previous study showed that there was no significant difference in erectile function when MSCs were implanted in the MPG or the corpus cavernosum.^37^ However, MSC implantation in the MPG promoted nerve regeneration, while MSC implantation in the corpus cavernosum prevented degeneration of SM.^38^ The leading cause of fibrosis in the corpus cavernosum after BCNI is neuronal Wallerian degeneration in the MPG and the CN.^39^ Transplantation of MSCs around the MPG may result in secretion of neurotrophic factors to promote nerve regeneration at the CNI site.^38^ In this study, we transplanted GM‐ADSCs around the MPG. The expression of GFP and RFP (the marker transfected with ADSC) was increased in the MPG 2 weeks after transplantation, which indicated higher levels of VEGF and GDNF in the MPG, and suggested that the graft was integrated into the tissue, resulting in CN repair. We also found GM‐ADSC residues in the penis, which suggested that the implanted cells around the MPG may have reached the target organ through blood flow. In addition, VEGF and GDNF were expressed at higher levels in the penis in the GM‐ADSC groups than in the PBS group. Interestingly, we found that in the sham and PBS group, the immunoreactivity score of VEGF and GDNF in MPG was lower than those of other different types of ADSC. We speculated firstly that VEGF and GDNF could not be continuously and effectively increased at the site of nerve injury, and the short half‐life of them in both normal and injury tissues, thus may let them be hard to detect in the Sham/ PBS group, resulting in a low immunoreactivity score.^40^, ^41^ ADSC has a unique inherent paracrine function which has been demonstrated to be capable of secreting GDNF and VEGF before,^12^, ^42^ and genetically modified ADSC in this research sustained focal released of the desired neurotrophin, thus may increase immunoreactivity score of VEGF and GDNF in ADSC‐V, ADSC‐G and vehicle cohorts than Sham/ PBS group. In addition, the immunoreactivity score of VEGF and GDNF in ADSC‐G&V was higher than those of ADSC‐V and ADSC‐G group, which may cause by following two reasons: First, GDNF and VEGF may have a synergistic effect to promote increased expression of each other, which is consistent with previous research results^43^; Second, VEGF and GDNF together may repair the cavernous nerve more effectively, then GDNF and VEGF produced by the penis could more fluently retrogradely transported to MPG and may further assist nerve regeneration.^44^ The most significant improvement in the functional and tissue endpoints of ADSC‐G&V in this study may in turn explain this hypothesis. The parasympathetic branch of the CN is the main nerve that regulates penile erection.^4^ Studies have shown that the expression of the GDNF receptor (GFRα1) does not decrease following CN injury, and GFRα1 is expressed in both sympathetic and parasympathetic nerves, which suggests that GDNF may be an effector of CN repair.^4^, ^45^ Importantly, GDNF produced by the penis can be retrogradely transported to the pelvic nerve for neuroprotection.^44^ In addition to its role of angiogenesis, VEGF can promote axonal regeneration and protect muscle fibres from degeneration.^46^, ^47^ Studies have shown that VEGF can stimulate SC migration and neovascularization after sciatic nerve transection.^48^ Our in vitro results showed that chemotaxis of primary SC in the ADSC‐G&V group was more pronounced than that in the ADSC‐G group, which suggested that VEGF may have enhanced SC migration in cooperation with GDNF. Therefore, we hypothesized that VEGF and GDNF from GM‐ADSCs transplanted around the MPG and in the penis may synergistically promote CN regeneration and protect the penile nitrogen nerve.

S100β, a neuronal myelin sheath marker, was markedly increased in the MPG in the ADSC‐G&V group compared with the other intervention groups. Neurofilament‐H, a component of the mature neuronal cytoskeleton, was also significantly increased in the MPG in the ADSC‐G&V group, which indicated that ADSC‐G&V promoted nerve regeneration and myelin sheath formation better than cells that secreted VEGF or GDNF alone. We also evaluated changes in the penile erectile nerves. Studies have shown that nNOS‐positive neurons could represent penile projection neurons,^49^ which is an important element involved in penile erectile function. As such, we performed immunofluorescence staining of penile nNOS to identify cavernous nerves. We found that the content of nNOS in the penis in the ADSC‐G&V group was significantly greater than that in the other intervention groups, which was consistent with the neuroprotective effects observed in the MPG.

Studies have shown that the corpus cavernosum began to show obvious fibrosis about 1 week after BCNI, and fibrosis progressed over time.^9^ Although the fibrotic response is likely protective in nature, progression of fibrosis can result in irreversible cellular dysfunction or organ failure.^30^ Therefore, early penile rehabilitation is crucial. We evaluated penile erectile function and fibrosis of the corpus cavernosum 2 weeks after rat BCNI. The PBS group exhibited obvious fibrosis in the cavernous cavernosum (lower SM/collagen ratio), and desmin, a marker of smooth muscle was expressed at very low levels, which suggested that the corpus cavernosum underwent severe fibrosis within 2 weeks of BCNI. These findings were similar to those in previous studies.^9^, ^33^ However, in the ADSC‐G&V group, the SM/collagen ratio and the expression of desmin in the corpus cavernosum were significantly higher than those in the PBS group, which indicated that progression of penile fibrosis may be prevented.

Neurapraxia caused by CNI can induce temporary or persistent hypoxia of the penis, which can result in upregulation of TGF‐β1 to promote cavernous fibrosis.^50^ To further investigate hypoxia in the corpus cavernosum, we performed immunofluorescence staining of the hypoxia marker HIF‐1α. As expected, we found that HIF‐1α was highly expressed in the PBS group, which indicated significant hypoxia compared to the other groups. However, HIF‐1α was expressed at very low levels in the sham group, and the expression of HIF‐1α in the ADSC‐G&V group was significantly lower than that in the other intervention groups, which was consistent with the observed changes in the SM/collagen ratio. Increased endothelial content in the corpus cavernosum is an important component of functional recovery of erectile function. The expression of the endothelial cell–specific marker RECA‐1 in the ADSC‐G&V group was significantly higher than that in the PBS group, which was consistent with improved erectile function. To further characterize the mechanisms of improvement of penile fibrosis in this study, we evaluated the fibrosis pathway p‐LIMK2/p‐cofilin. This pathway was significantly downregulated in the penises of animals subjected to CNI that received GM‐ADSC therapy, which was consistent with previous studies.^32^ Furthermore, the ADSC‐G&V group reduced the expression of this pathway to the greatest extent.

A leading cause of poor nerve regeneration is transient expression of growth‐related genes.^51^ Studies have shown that genetic delivery of neurotrophic factors may provide therapeutic benefits.^22^ The half‐life of neurotrophic factors is short, and they are limited by low bioavailability when administered exogenously. Therefore, genetic modification to induce sustained release of neurotrophic factors has been shown to be more effective.^41^

Our study had some limitations. Our intervention was administered immediately after BCNI. The therapeutic effects of GM‐ADSC administered at different time points following BCNI should be evaluated. Furthermore, additional optimization of delivery of growth factors is needed to ensure efficacy and safety.

In conclusion, this study supported the hypothesis that GM‐ADSCs that overexpressed VEGF and GDNF could repair CN speedily, prevent penile fibrosis and protect the integrity of penile local functional tissues, resulting in recovery of erectile function. Transplantation of ADSCs that co‐expressed VEGF and GDNF‐induced synergistic effects, resulting in improved recovery compared to ADSCs that expressed VEGF or GDNF alone. The results of this study may provide a new and promising approach to rapid amelioration of ED caused by CNI.

## CONFLICT OF INTEREST

The authors declare that the research was conducted in the absence of any commercial or financial relationships that could be construed as a potential conflict of interest.

## AUTHOR CONTRIBUTIONS

HBW and WDY participated in study design; WDY and ZHC and XLM performed research; WDY and HBW involved in manuscript preparation; JFF and XOY involved in methodology and data analysis.

## Data Availability

The data that support the findings of this study are available from the corresponding author upon reasonable request.
